# The self-reactive FVIII T cell repertoire in healthy individuals relies on a short set of epitopes and public clonotypes

**DOI:** 10.3389/fimmu.2024.1345195

**Published:** 2024-03-06

**Authors:** Valeria Porcheddu, Gautier Lhomme, Rémi Giraudet, Evelyne Correia, Bernard Maillère

**Affiliations:** Université de Paris-Saclay, Commissariat à l'énergie atomique et aux énergies alternatives (CEA), Institut national de recherche pour l’agriculture, l’alimentation et l’environnement (INRAE), Département Médicaments et Technologies pour la Santé, Service d’Ingénierie Moléculaire pour la Santé (SIMoS), Gif-sur-Yvette, France

**Keywords:** CD4 T cell, TCR - T cell receptor, hemophilia A, FVIII = factor VIII, antigenicity, epitope mapping, self-reactivity, thymic selection

## Abstract

Non-mutated FVIII-specific CD4 T cell epitopes have been recently found to contribute to the development of inhibitors in patients with hemophilia A (HA), while auto-reactive CD4 T cells specific to FVIII circulate in the blood of healthy individuals at a frequency close to the foreign protein ovalbumin. Thus, although FVIII is a self-protein, the central tolerance raised against FVIII appears to be low. In this study, we conducted a comprehensive analysis of the FVIII CD4 T cell repertoire in 29 healthy donors. Sequencing of the CDR3β TCR region from isolated FVIII-specific CD4 T cells revealed a limited usage and pairing of TRBV and TRBJ genes as well as a mostly hydrophobic composition of the CDR3β region according to their auto-reactivity. The FVIII repertoire is dominated by a few clonotypes, with only 13 clonotypes accounting for half of the FVIII response. Through a large-scale epitope mapping of the full-length FVIII sequence, we identified 18 immunodominant epitopes located in the A1, A3, C1, and C2 domains and covering half of the T cell response. These epitopes exhibited a broad specificity for HLA-DR or DP molecules or both. T cell priming with this reduced set of peptides revealed that highly expanded clonotypes specific to these epitopes were responsible individually for up to 32% of the total FVIII repertoire. These FVIII T cell epitopes and clonotypes were shared among HLA-unrelated donors tested and previously reported HA patients. Our study highlights the role of the auto-reactive T cell response against FVIII in HA and its similarity to the response observed in healthy individuals. Thus, it provides valuable insights for the development of new tolerance induction and deimmunization strategies.

## Highlights

- 18 immunodominant and immunoprevalent HLA-DR/DP restricted epitopes elicit the majority of the CD4 T cell response in healthy individuals.
- TCR CDR3β sequencing of the auto-reactive FVIII T cell repertoire shows public clonotype, identified in multiple Haemophilic A patients.

## Introduction

Factor VIII (FVIII) is an essential self-protein involved in the coagulation cascade. The lack of FVIII in the blood circulation leads to the inability to coagulate and therefore to life-threatening spontaneous hemorrhagic bleeds. The severity of hemophilia A (HA) is determined by the residual FVIII activities of circulating endogenous FVIII, which result from mutations and deletions in the FVIII-encoding F8 gene (1, 2). Recombinant or plasma-derived FVIII remains a life-saving protein drug for HA patients. Although new hemostatic agents, bypassing the need for FVIII replacement, are now available, spontaneous bleeds may still occur in individuals under prophylaxis non-FVIII therapies, leading to the need for FVIII injections (3, 4). However, the development of neutralizing anti-FVIII antibodies, known as “inhibitors”, can complicate or preclude effective FVIII replacement therapy (5). The immunological mechanisms behind the immunogenicity of pro-coagulant FVIII remain unclear (6). The involvement of CD4 T cells in the onset of the immune response against FVIII was first suggested in the 1980s on HIV-positive HA patients, who showed a reduction in inhibitor levels as the CD4 T cell population declined (7). FVIII-specific antibodies are class-switched IgG and IgA antibodies (8, 9), as is typical for CD4 T cell-driven immune responses (10, 11). The incidence of inhibitors is indeed higher in patients with no endogenous FVIII production than in patients with mild/moderate forms of the disease who have residual FVIII in their blood. This suggests that the foreignness of FVIII and the residual expression of mutated FVIII in HA is an important parameter controlling FVIII immunogenicity, which might result from a deficiency in the deletion of CD4 T cells in the thymus. However, recent *in vitro* studies have demonstrated how in HA subjects the T cell response is triggered not only by epitopes located in the mutated sequences of the protein but mostly by fully self-FVIII sequences (12). This suggests that lack of tolerance is not only limited to the foreign parts of the FVIII molecule but extended to non-mutated FVIII sequences (13, 14). Additionally, inhibitor development in non-HA individuals can occur as a rare but serious autoimmune reaction (15, 16). Multiple studies demonstrated the presence of low-affinity non-neutralizing anti-FVIII antibodies in healthy individuals (17, 18) as well as the existence of a specific FVIII CD4 T population (19). These findings strongly suggest that an immune response against this endogenous protein is ongoing in healthy individuals under physiological conditions. Understanding the immune response to FVIII in healthy individuals can provide new insights into the mechanisms underlying inhibitor development and potentially inform new tolerance induction strategies in the context of hemophilia A and also give a new perspective on the central tolerance mechanism to self.

## Materials and methods

### Proteins and peptides

Hemocyanin from keyhole limpet (KLH) was purchased from Sigma-Aldrich (Saint-Quentin-Fallavier, France). rFVIII Kogenate was a kind gift from Dr. Pedro Paz and Dr. Jeanette Lo (Bayer, San Francisco, CA, USA). Potential T cell epitopes were selected from the full-length FVIII (UniProt ID: P00451) using NetMHCIIpan 4.0 as described in **Supplementary Table S1**. The corresponding peptides were synthesized by Proteogenix Science (France). Ovalbumin was purchased from Sigma-Aldrich (France).

### Enrichment of specific T cell lines

PBMCs were purified from the whole blood of anonymous healthy donors, who gave informed consent (Etablissement Français du Sang, Rungis, France), using Ficoll-Paque®. Monocyte-derived dendritic cells (DCs) were produced from cells isolated using Percoll gradient by 5 days of culture in AIM-V medium (Invitrogen, Villebon-sur-Yvette, France) supplemented with 1,000 U/ml recombinant human (rh) IL-4 and rhGM-CSF (both from R&D Systems, Lille, France). Mature DCs were produced by two supplementary days of culture in the presence of LPS (1 µg/ml) and R848 (10µg/ml). CD4 T cells were isolated from autologous PBMCs by positive selection using magnetic labeling with anti-CD4 mAbs conjugated to magnetic microbeads followed by magnetic cell sorting, as recommended by the manufacturer (Miltenyi Biotec, Bergisch Gladbach, Germany). Immature DCs were loaded overnight at 37˚C with either 0.2 µM of rFVIII, 0.2 µM of ovalbumin, or 0.25 µM of KLH in 96-well U-bottom plates, while mature DCs were used for FVIII peptides (10 ug/ml). CD4 T cell lines were generated *in vitro* in IMDM (Invitrogen) supplemented with 10% human serum, 0.24 mM glutamine, 0.55 mM asparagine, 1.5 mM arginine (all amino acids from Sigma-Aldrich), 50 U/mL penicillin/50 μg/mL of streptomycin (Invitrogen), 20 IU/mL human IL-2, and 17 IU/ml human IL-7 (premium-grade; Miltenyi Biotec) by three once-per-week rounds of stimulation of CD4 T cells (200,000 per well) with autologous DCs (20,000 per well). 10,000 lymphocytes were collected at day 19 from each T cell line (expanded T cells contained in one well) and their specificity was assessed by using IFN-g ELISPOT.

### IFN-γ ELISPOT assay

T cells were incubated with autologous DCs alone (control) or with DCs incubated with rFVIII (0.2 µM), ovalbumin (3 µM), and KLH (1 µM) or PBMCs for FVIII peptides (10 µg/ml) in MultiScreen 96-well plates (Merck Millipore) previously coated with 2.5 µg/mL of anti-human IFN-γ monoclonal antibody (mAb; 1-D1K; Mabtech). The T cell lines and APCs were plated at equal number for both stimulated and unstimulated control conditions. After overnight incubation, spots were revealed using 0.25 µg/mL of biotinylated anti-human IFN-γ mAb (7-B6-1; Mabtech) in phosphate-buffered saline/bovine serum albumin 1%, extravidin-phosphatase (dilution 1:3,000 in phosphate-buffered saline/Tween20 0.05%/bovine serum albumin 1%; Sigma-Aldrich), and nitro blue tetrazolium/5-bromo-4-chloro-3-indolyl phosphate-toluidine salt (Sigma-Aldrich). The spot number was determined by using the ELISPOT Reader System (Autoimmun Diagnostika, Ebinger, Germany). HLA restriction was assessed using monoclonal antibodies (10 μg/ml) specific to HLA-DP (B7/21), HLA-DQ (SPVL3), or HLA-DR (L243).

### Sorting of FVIII-specific T cells by cytometry

Positive antigen-specific T cell lines determined by using the ELISPOT assay were pooled together at day 21, after 24 h of resting in IMDM medium and with the absence of cytokine stimuli. The pooled T cell lines were re-stimulated overnight with autologous DCs alone (control) or with DCs previously loaded with either 0.2 µM of rFVIII, 0.2 µM of ovalbumin, or 0.25 µM of KLH. Following 24 h of stimulation, cells were harvested in cell staining buffer (phosphate-buffered saline/EDTA 2 mM/bovine serum albumin 0.5%) and then stained for CD4 (Vio-Blue, clone REA623, Miltenyi Biotec) and activation-induced markers CD134 (OX-40) (PE anti-human, clone Ber-ACT35, Biolegend) and CD137 (4-1BB) (APC anti-human, clone 4B4-1, Biolegend). The CD4/CD134/CD137-positive population for each condition was sorted by flow cytometry (FACSARIA III; Becton Dickinson, San Jose, CA, USA).

### RNA extraction and library preparation

RNA was extracted from antigen-specific T cells using the RNeasy Plus Mini Kit (Qiagen), and sample RNA was reverse-transcribed using a TCRB chain constant region reverse primer with Superscript IV First-Strand Synthesis System (Invitrogen). cDNA was column-purified with the Oligo Clean and Concentrator Kit (Zymo Research). cDNA was used as a template for PCR with KAPA2G Fast Multiplex Mix (Roche). Forward TCRB FR3 primers were used with a single nested TCRB constant region reverse primer. A total of 20 cycles for NGS library preparation for the PCR1 and 20 cycles of PCR2 were performed on the PCR1 product to incorporate sample barcodes and Illumina sequencing adaptors using Herculase II Fusion DNA Polymerase (Agilent). Specific principles and methodology have been previously reported (20). The PCR2 product underwent a run of agarose gel electrophoresis. Bands at 300 pb were purified using a GeneJET purification kit (Thermo Fisher) and quantified using the Qubit dsDNA HS Assay Kit (Thermo Fisher Scientific).

### Bulk sequencing and TCR data analyses

The samples were sequenced on an Illumina NextSeq 500 with 15% of PhiX control v3 (Illumina). All data were aligned using the MiXCR v4.2.0 tool (https://github.com/milaboratory/mixcr). The high-quality reads were further assembled into clonotypes, correcting for PCR and sequencing errors. Shared clonotypes between the unstimulated and antigen-stimulated samples were removed. The downstream analysis was performed with MIXCR, “Immunarch” R package, and in-house R https://www.ncbi.nlm.nih.gov/geo/query/acc.cgi?acc=GSE253521script. All data are accessible on the NCBI GEO database (accession number: GSE253521; https://www.ncbi.nlm.nih.gov/geo/query/acc.cgi?acc=GSE253521).

### Graphical illustrations

Graphical illustrations were made within FlowJo 10, GraphPad Prism 8.3.0, the “Immunarch” R package, and BioRender (biorender.com). VJ chord diagram was created using “circlize” CRAN package.

## Results

### rFVIII and ovalbumin induce *in vitro* T cell response with similar amplitude and diversity in healthy donors

**Figure 1:**
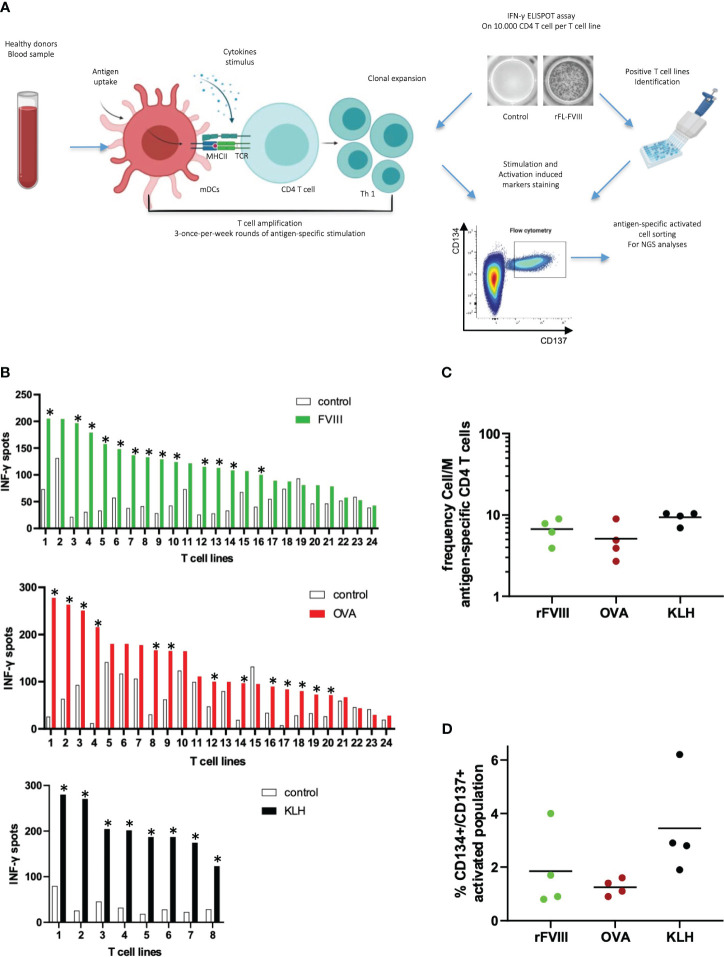
Characterization of specific T cell lines for rFVIII and ovalbumin in healthy donors. T cell lines were generated by a co-culture of antigen-loaded DCs with CD4 T cells for 3 weeks. Antigen-specific T cell lines were identified by IFN-γ Elispot and pulled for a supplementary activation with and without antigen. After 24 h, CD134/CD137 cells were sorted and their TCR CDR3β sequenced **(A)**. Donor #1180: means of IFN-γ spots per T cell line tested in duplicate for antigen and unloaded DCs control. CD4 T cell lines were considered specific when a spot count was twofold higher in the presence of the protein or peptide than in its absence, with a minimum difference of 25 spots. The asterisks identify positive T cell lines: 13/24 for rFVIII, 13/24 for Ova, and 8/8 for KLH control **(B)**. CD4 T cell precursor frequencies were estimated using the Poisson distribution according to the following formula: frequency = -Ln [(number of nonspecific CD4 T cell lines/total number of CD4 T cell lines seeded)]/(number of CD4 T cells per well) **(C)**. Positive T cell lines identified for each antigen were pooled together, split, and stimulated overnight for two independent conditions: stimulated with autologous FVIII-loaded DCs and autologous unloaded DCs as unstimulated control. The level of activation was calculated as percentage of CD134/CD137 double-positive gate. The graph shows the mean percentage of double-positive CD134/CD137 sorted cells on the total CD4 population for each of the four donors tested. Activation level % of the negative unstimulated control was removed from each stimulated sample **(D)**.

**Figure 2:**
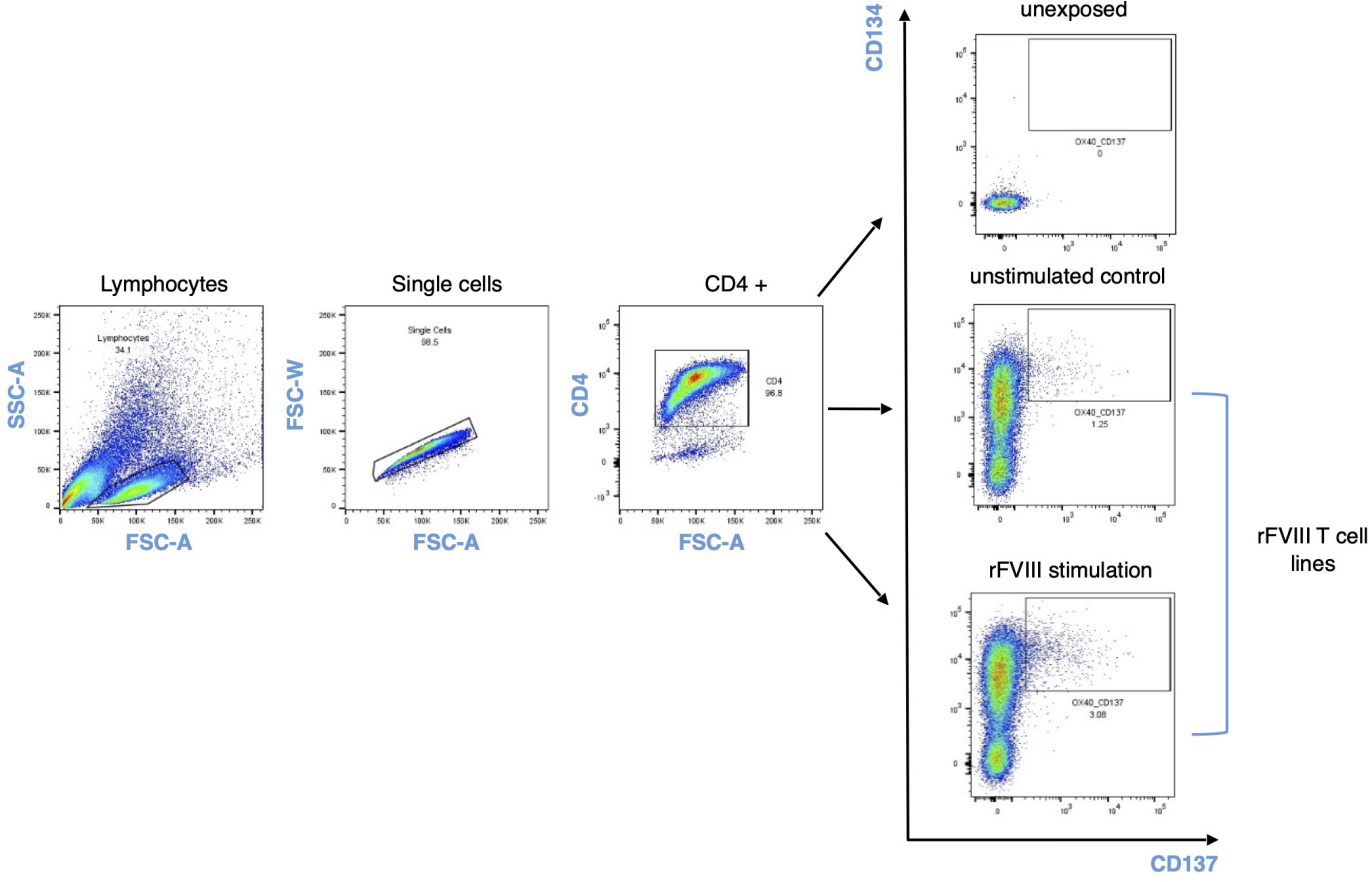
AIM CD137/CD134 gating strategy. Cells were stained and sorted based on CD134/CD137 double-positive activation-induced markers 24 h after rFVIII stimulation and unstimulated control for each donor in the cohort. The unexposed sample refer to *ex vivo* unstimulated CD4 T cell population and their expression of activation-induced markers.

**Figure 3:**
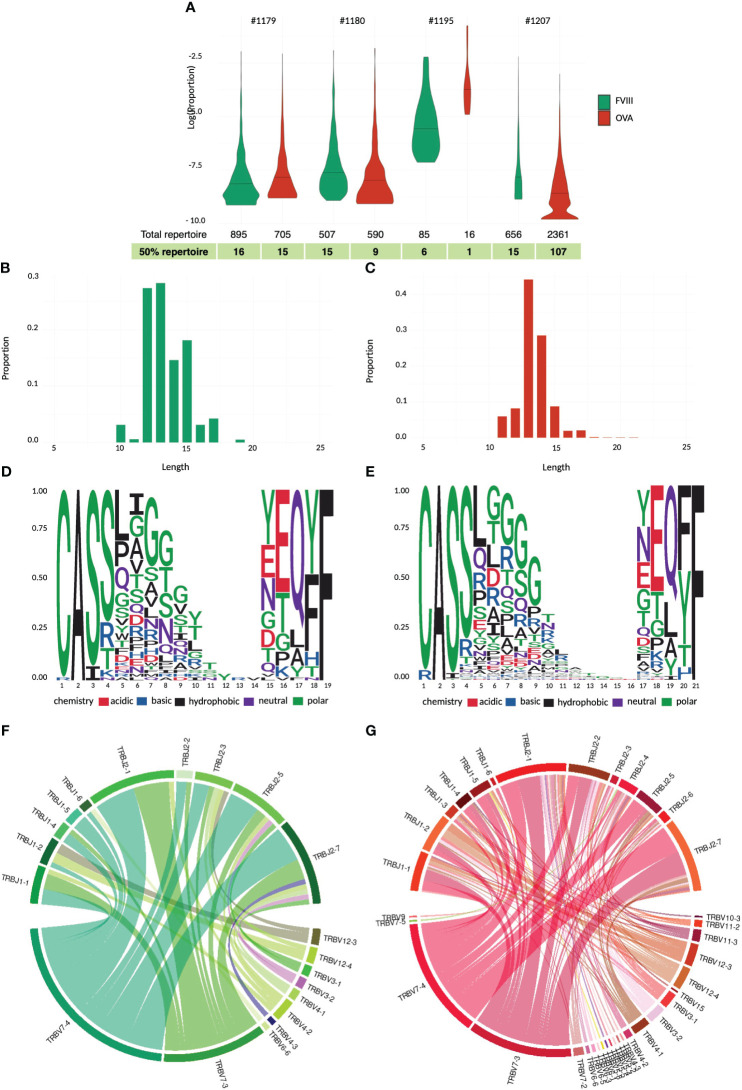
TCR CDR3β repertoire analyses and comparison for rFVIII and ovalbumin. Violin plot of total repertoire for FVIII and ovalbumin plotted as log(proportion) of each clonotype sequence for individual donors tested. The quantile bar defines 50% of the repertoire distribution **(A)**. CD3 length distribution of the highly expanded clonotypes in the top 50% of the FVIII **(B)** and ovalbumin repertoire **(C)**. Sequence logo representation of complete alignment sequence dataset of the top 50% FVIII **(D)** and ova repertoire **(E)**. Relative frequency of each amino acid in each CDR3β position calculated on the entire antigen dataset. The residues are color-coded according to their chemical properties. Changes in variable-joining segment pairing in the CDR3 junctions between FVIII **(F)** and ovalbumin **(G)** top 50% clonotypes. The chord diagram is used for visualization; ribbons connecting segment pairs are scaled by corresponding V–J pair frequency.

Firstly, we aimed to characterize the T cell response to rFVIII in vitro using chicken ovalbumin (Ova) as a non-self-protein benchmark. Given the low frequency of FVIII-specific T cells in healthy donors, we enriched the CD4 population in vitro before analyzing their specificity and sorting them for TCR CDR3β sequencing (**Figure 1A**). CD4 T cells from four healthy donors were stimulated by three rounds of stimulation with DCs loaded with FVIII, Ova, or KLH as a positive control. Antigen specificity was assessed using the IFN-γ ELISPOT assay. All four donors responded to KLH, while only a portion of the T cell lines raised against rFVIII and Ova showed a specific response, indicating the limited dilution conditions of specific T cells at the start of the culture (19) (**Figure 1B**). FVIII and ovalbumin generated 69 and 58 specific T cell lines, respectively, among 96 T cell lines tested for each antigen. As demonstrated previously (21), the distribution of the antigen-specific CD4 T cells at the initiation of our assay follows a Poisson distribution. This allows us to calculate the mean frequency of antigen-specific CD4 T cells. Interestingly, the number of precursor frequencies able to recognize specifically FVIII was comparable to the response obtained for the non-self-protein ovalbumin, namely, 6 and 5 cells/M (**Figure 1C**). The identified positive T cell lines were re-stimulated overnight with the respective antigen of specificity or unloaded DCs as a negative control. The cells were then stained for T cell receptor (TCR)-dependent activation-induced markers (AIMs) CD137 (4-1BB) and CD134 (OX-40). The activation percentages obtained were comparable, with a mean of 1.85% for FVIII and 1.25% for ovalbumin, and the variability across the four tested donors ranged from 0.8% to 4.0% of activation for FVIII and 0.9% to 1.6% for Ova (**Figure 1D**). Antigen-specific CD4 T cells (CD134/CD137 double-positive cells) (**Figure 2**) and the corresponding unstimulated control samples were thus sorted and their TCR CDR3β sequenced (**Figure 3A**). Within individual donors, the total repertoire distribution among FVIII and Ova samples was generally similar. The average number per donor of detected clonotypes was 535 and 918, for a total of 2,143 and 3,672 clonotypes detected for FVIII and Ova. Using the Gini-Simpson index as a measure of T cell repertoire diversity and clonotype abundance, Ova and FVIII show a very similar polyclonal distribution among the donors tested, 0.95 and 0.98, respectively. Interestingly, for FVIII, on average 13 clonotypes are covering 50% of the total repertoire and 33 clonotypes instead for Ova. To investigate the CDR3β characteristics of these highly expanded clonotypes, the following analysis will refer to the clonotypes covering the top 50% of the total repertoire for both antigens. β-CDR3 sequences specific for FVIII had lengths ranging from 10 to 19 aa, with a preferential length between 12 and 13 aa (**Figure 3B**). While those for Ova had lengths ranging from 11 to 21 aa, with a preferential length of 13 aa (**Figure 3C**). CDR3 sequences of different lengths were aligned using “MUSCLE” (22). The CDR3β motifs showed no strong over-representation of specific aa, except for a slightly higher prevalence of glycine residues. In the FVIII CDR3β motif, we observed an enrichment in hydrophobic, polar, and neutral aa, where alanine, leucine, threonine, proline, and valine were the most present in the core sequence (**Figure 3D**). On the other hand, Ova shows a prevalence of polar, basic, and neutral aa composition, with arginine, serine, asparagine, glutamine, and tyrosine highly present (**Figure 3E**). Concerning the V motif, FVIII T cell clones show a narrow gene usage signature, predominantly represented by TRBV7-4 (60%) and TRBV7-3 (25%). Similarly, TRBJ1-2 and TRBJ2-7 are the genes encoding 50% of the total FVIII repertoire, followed by TRBJ2-5 (16%) and TRBJ1-1 (9%) (**Figure 3F**). On the other hand, ovalbumin showed a slightly wider VJ usage and less representative gene pairing. Among the TRBV, TRBV7-4 accounts for 35% of expression frequency among the total repertoire, followed by TRBV7-3 (34%), TRBV4-1 (7%), and TRBV12-3 (8%). The TRBJ genes showed a similar usage distribution, with TRBJ2-7 being expressed on 25% of the total repertoire and TRBJ2-1 (18%), TRBJ1-1 (11%), TRBJ2-5 (9%), TRBJ1-2 (8%), and TRBJ2-2 (8%) (**Figure 3G**).

### Large-scale mapping of CD4 T cell epitopes of rFVIII in healthy individuals

**Figure 4:**
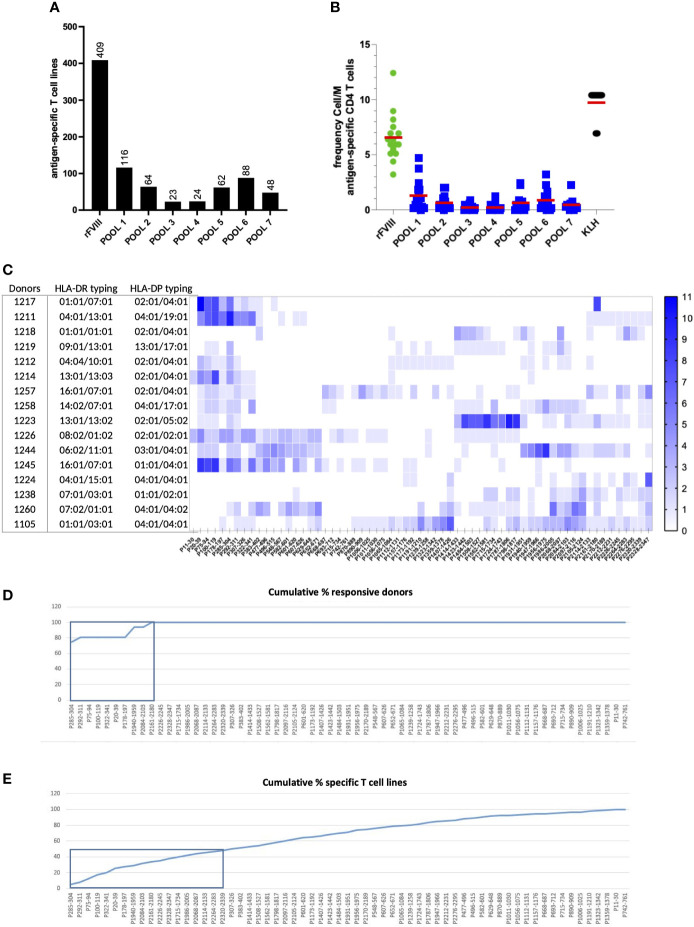
Large-scale mapping of CD4 T-cell epitopes of recombinant full-length FVIII in healthy individuals. The T cell lines were generated by a co-culture of antigen-loaded DCs with CD4 T cells for 3 weeks. Their response to seven pools composed of the 63 selected peptides and against rFVIII was assessed in the first ELISPOT. **(A)** Cumulative sum of the specific T cell lines raised across all donors against rFL-FVIII and the seven pools of FVIII peptides. **(B)** Frequency of the response cells/M for all the conditions tested among the 16 healthy donors. On the second ELISPOT, we then evaluated the response of the positive T cell lines against all the single peptides present in the reactive pools. **(C)** Heat map intensity response to all the 63 FVIII peptides tested per HLA-typed donor; the color code indicates the number of positive T cell lines identified per peptide tested. **(D)** Cumulative percentage of responding donors across all the 63 FVIII peptides tested, with 100% of the donor coverage reached within the response obtained by nine T cell epitopes. **(E)** The cumulative percentage of specific T cell lines identified demonstrated that 50% of the total T cell response to FVIII is elicited by 18 promiscuous immunodominant T cell epitopes.

Based on the T cell epitope prediction software NetMHCpan4.0 (23), we selected 63 20-mer peptides that are predicted for their ability to bind promiscuously, with a percentile score below 10%, at least four HLA class II molecules from a selection of 15 preponderant HLA-DR alleles in our panel (as shown in ST1). The predicted peptides spread all along the full-length FVIII sequence and account for more than 50% of its amino acid coverage. A total of 16 healthy donors with unrelated HLA typing (ST2) were used as sources of T cells to identify FVIII-specific CD4 T cell epitopes. Specific T cell lines were generated by *in vitro* T cell culture using FVIII and KLH. The peptide specificity of the T cell lines raised against FVIII was assessed by two independent IFN-γ ELISPOT using peptide pools and individual peptides. All donors responded to rFVIII with a total of 409 identified rFVIII-specific T cell lines (**Figure 4A**). The mean of CD4 T cell precursors was 6.5 cells/M (**Figure 4B**). Each of the 63 FVIII peptides induced a T cell response in at least one of the donors tested, among the 16 healthy donors tested, with 872 peptide-specific T cell lines identified (**Figure 4C**). The CD4 T cell epitopes identified were ranked based on their donor coverage capacity (**Figure 4D**) and response intensity (**Figure 4E**). Furthermore, 18 out of 63 FVIII T cell epitopes were responsible for 50% of the total T cell response and 100% of the donor coverage. These immunoprevalent epitopes are located in the A1, A3, C1, and C2 domains of the FVIII protein (ST3). Furthermore, four independent healthy donors were used as sources of T cells to characterize the HLA restriction and to assess the reactivity of T cell lines generated with the 18 peptides toward rFVIII. T cell lines were amplified *in vitro* for 28 days through stimulation with the pool of 18 peptides and then tested through IFN-γ ELISPOT. All four healthy donors tested responded to the pool of 18 FVIII peptides selected, with a total of 87 specific T cell lines identified. Moreover, 16 out of 18 FVIII peptides in the pool generated a specific T cell response among the four donors. A total of 14 of the T cell peptides were tested for recognition of rFVIII-loaded on autologous DCs, and it was confirmed that they were part of the naturally processed FVIII peptides. HLA restriction was assessed by introducing anti-HLA-DR, anti-HLA-DP, and anti-HLA-DQ antibodies. None of the peptides tested was HLA-DQ restricted. Eight peptides were exclusively HLA-DR-restricted, one peptide was HLA-DP, and seven were promiscuously presented on both HLA-DR and HLA-DP molecules (ST4). These data confirmed the *in silico* binding affinity prediction for both HLA-DR and DP molecules (ST6). Furthermore, the 18 FVIII peptides selected were ulteriorly tested in an *in vitro* binding assay to six HLA-DRB1 molecules (as described in ST5).

### Public TCR sequences specific for 18 FVIII epitopes are inducing the majority of the FVIII T cell response

**Figure 5:**
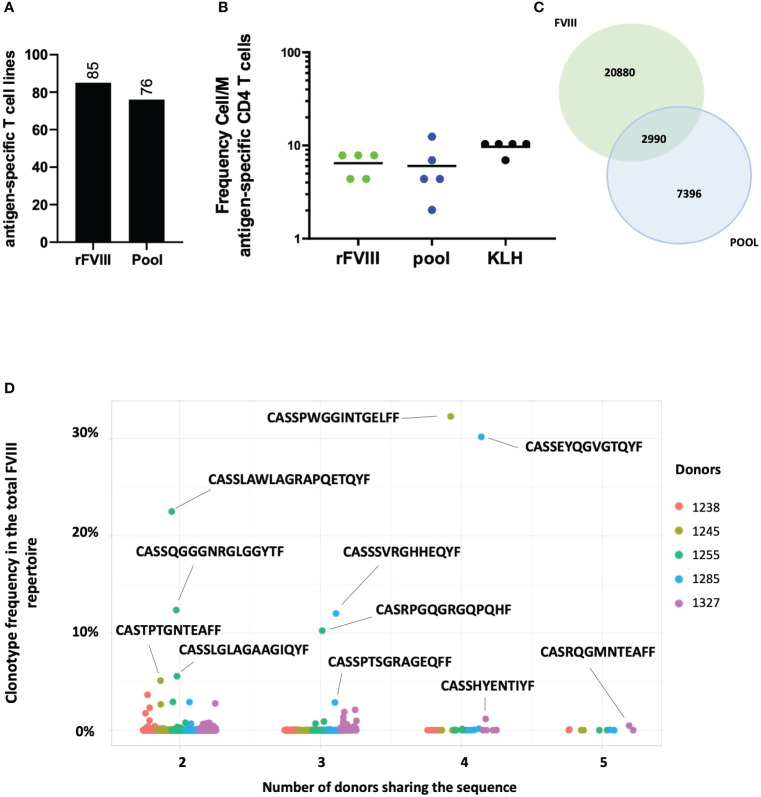
Repertoire analyses between rFVIII and the pool of 18 immunodominant peptides. Number of cumulative T cell lines identified through the INF-γ ELISPOT assay, specific for rFVIII and the pool identified among the five donors tested **(A)**. Frequency of CD4 T cell precursors Cell/M. **(B)** Venn diagram showing the 2,990 shared TCR sequences between FVIII and pool samples on the total number of specific clonotypes found for both FVIII and pool across the five donors tested **(C)**. Clonotype frequency in the total FVIII repertoire (y axis) of the 178 public TCR sequences between pool and FVIII samples by the number of donors sharing the same sequence (x axis) among the five donors tested. Each dot represents a single clonotype and its frequency by donor (as shown in the color legend) **(D)**.

Ultimately, we aimed to characterize the T cell response against the 18 FVIII epitopes selected and compare it to the response to the full-length protein. Five healthy donors were used as sources of T cells. The T cells were amplified *in vitro* for 28 days as previously described. For each donor, 24 T cell lines were stimulated with a pool of the 18 FVIII epitopes, 24 T cell lines with rFVIII, and eight T cell lines with KLH simultaneously. Specificity was then assessed by using IFN-γ ELISPOT. All donors tested responded to the pool of 18 FVIII epitopes and rFVIII. A total of 76 T cell lines specific for the pool and 85 specific for rFVIII were identified (**Figure 5A**). Calculation of the antigen-specific precursor frequency in this experiment confirmed again 6.4 cells/M for rFVIII and 6 cells/M for the pool (**Figure 5B**). To characterize TCR sequences responding to the pool and their distribution within the total rFVIII repertoire, specific T cell lines were sorted based on AIM, and TCR CDR3β bulk sequencing was performed. We found 2,990 shared expanded sequences between the FVIII and pool repertoire among the five donors (**Figure 5C**). Surprisingly, 178 of these sequences are public clonotypes shared among multiple HLA-unrelated donors, as shown in **Figure 5D**, accounting individually for up to 32% of the total FVIII repertoire.

## Discussion

Our study aims to characterize the diversity and the epitope specificity of the self-reactive FVIII CD4 T cell repertoire in healthy individuals to implement our understanding of the T cell response to FVIII.

Investigation of low-frequency antigen-specific CD4 T cells from donors who have never been injected with the antigen represents a challenge and requires specific modalities in the design of the T cell assay. Our method involves weekly *in vitro* stimulation of the T cell population with the antigen of interest. This allows us to enrich the cellular population in rare clonal expanded antigen-specific T cells (21, 24–28). The culture conditions trigger the skewing of the CD4 T cells toward a Th1 phenotype, allowing the detection of antigen-specific T cells through IFN-γ production. While this approach does not permit phenotype analyses, it offers the opportunity for cell frequency estimation (19), epitope identification, and TCR analyses through CDR3β sequencing of AIM-expressing T cells. CD137 (4-1BB) and CD134 (OX-40) used in this study are cell surface markers, which are specifically induced by TCR engagement and expressed by antigen-specific T cells upon antigen recognition (29–33).

T cell immune response is strongly dependent on the diversity and availability of TCR receptors affine to a given antigen, resulting from the combinatorial diversity of the TCR repertoire of each individual. The ability of the adaptive immune system to cover such a huge diversity (34) depends on the VDJ segment rearrangement and through pre-TCR/self-pMHC interaction during β-selection (35–41). Indeed the T cell repertoire is selected in the thymus against self (42), where the self-pMHC-TCR level of reactivity, within a very strict threshold (43), determines deletion or selection and phenotype fate (44, 45). Therefore, the self-immunopeptidome presented by thymic APCs shapes the naive T cell size and diversity. In the present study, we confirm the previous observation that high-frequency FVIII T cells circulate in the blood of healthy donors (19) with a frequency that is one log_10_ higher than pre-existing T cells specific for other immunogenic therapeutic proteins (46). One of the main hypotheses suggested to explain this self-reactivity is the insufficiency of intra-thymic FVIII expression. FVIII mRNA has been found in medullary thymic epithelial cells from healthy individuals (47–49). Further mRNA detection does not indicate the quantity of protein produced, which may not be sufficient to be widely displayed by APCs for T cell recognition. β, positive, and negative selection to self are happening in different thymic compartments, thymic cortex, and medulla respectively, and self-pMHC presentation is performed by different APCs (50–54). This creates a great variability between these compartments during the different phases of thymocyte development, and it might account for the escape of FVIII-specific CD4 T cells. Interestingly, the frequencies of T cells specific for Ova, which serves as a non-self-benchmark protein, were found comparable to those of FVIII. Ova is a well-known egg allergen in humans, and its non-self-origin implies a total absence of intra-thymic expression of this protein and therefore of T cell deletion by negative thymic selection. This equivalence in T cell precursor frequency between FVIII and Ova highlights the importance of FVIII-specific T cell escape and the impaired intra-thymic selection to FVIII. We further compared their clonotype distribution by sequencing the CDR3β of antigen-specific sorted T cells. The next-generation sequencing analysis of both repertoires shows a narrow VDJ usage signature and preferential VJ pairing for FVIII compared to Ova. The Ova-specific T cell repertoire exhibited a broader gene usage with a randomly distributed VJ pairing than the FVIII-specific T cell repertoire. Furthermore, the FVIII CDR3β motif shows an enrichment in highly hydrophobic amino acid, previously reported as a sign of CD4 T cell auto-reactivity (55).

Additionally, we conducted a large-scale T cell epitope mapping using a selected pool of peptides spanning more than 50% of the total FVIII sequence. The response against the potential epitopes selected was variable among the donors tested, regardless of their HLA-DR haplotypes. This highlights the large variability of the epitope-specific T cell repertoire and its role in shaping the response. Overall, 18 FVIII immunodominant and immunoprevalent epitopes located in the A1, A3, C1, and C2 domains were identified. These epitopes elicit the majority of the FVIII response in terms of the number of specific T cell lines and donor coverage. They are naturally promiscuously presented on both HLA-DR and HLA-DP molecules. HLA-DP restriction was confirmed by *in silico* netMHCIIpan4.0 prediction on HLA-DPB1*0401 and HLA-DPB1*0402 alleles. None of the T cell epitopes that we identified in the B domain are included in these 18 immunodominant peptides, suggesting that the B domain sequence may have a small impact on the immunogenicity of the FVIII protein, as demonstrated in several clinical trials (56–58). We do not exclude that other T cell epitopes may exist, especially if they are modified by post-translational modification such as glycosylation (59). As we did not determine the F8 haplotypes of the healthy donors tested, it is also possible that biologically active neutral mutations generate neo-epitopes that we could not detect with our strategy. We further investigated to which extent the response to the immunodominant pool of epitopes was representative of the full-length FVIII repertoire. The TCR sequence analysis revealed public clonotypes shared among donors in the FVIII and pool samples, which counted individually for up to 32% of the total FVIII repertoire. Three out of five donors in our study share one particular TCR sequence (CASSFDPLNTEAFF), and two out of five shared another TCR sequence (CASSFDQLNTEAFV) in the rFVIII and pool sample, both previously found to be specific for a peptide in the C2 domain included in our FVIII pool (http://tools.iedb.org/tcrmatch) (60). Multiple publications reported responses to the same C2 domain epitope in various HA patients carrying different F8 mutations (13, 61–65). It is noteworthy that the same public TCR sequence specific to this epitope has also been identified in one severe HA patient with persistent high-titer inhibitors, who failed ITI therapy (66). The patient presented a multi-exon deletion at positions 7 to 13 that eliminated almost the entire A2 domain-coding region. However, the T cell response was found to be predominantly directed against the C2-2212-2231 peptide, a fully self-sequence. These findings demonstrate the role of the auto-reactive T cell response against FVIII in HA and its similarity to the response observed in healthy individuals. The lack of TCR sequencing studies on HA patients limits public database resources and therefore further comparisons with our data. Overall, we conducted CDR3β bulk sequencing on a total of nine donors, four of which were stimulated with rFVIII and ovalbumin and five stimulated with rFVIII and the pool of selected immunodominant FVIII epitopes simultaneously. As previously shown, on average, 13 clonotypes per donor are sufficient to cover 50% of the total FVIII repertoire. Among the first four donors tested for FVIII and Ova, 53 highly expanded sequences cover half of the total FVIII clonotype distribution. Of these representative TCR sequences, 25 are public clonotypes shared among all nine HLA-unrelated donors tested and the pool of immunodominant FVIII epitopes repertoire, confirming once again the immunoprevalence of the epitopes selected which are representative of the major response to FVIII. To date, 15 FVIII T cell epitopes have been identified among HA patients across various publications (13, 61–70). All identified epitopes were included in the 63 FVIII peptides tested for their antigenicity in healthy individuals. Of the 18 immunodominant peptides selected in our study, six peptides had previously been described as T cell epitopes in multiple HA patients, while 12 are newly discovered T cell epitopes, as shown in **Table 1**. The findings suggest a comparable T cell repertoire that recognizes FVIII in both cohorts. This is at least partially independent of their F8 mutation. Further research may help elucidate any differences in immune response and potential implications for hemophilia A pathogenesis and treatment.

**Table 1:** Total of 18 immudominant and immunoprevalent FVIII peptides identified in healthy donors and comparison with previously identified FVIII T cell epitopes in hemophilia A (HA) patients.

Position	Sequence	FVIII domain	T cell response in HA patients
P285-304	VHSIFLEGHTFLVRNHRQAS	A1	/
P292-311	GHTFLVRNHRQASLEISPIT	A1	/
P75-94	DHLFNIAKPRPPWMGLLGPT	A1	/
P100-119	YDTVVITLKNMASHPVSLHA	A1	/
P322-341	LGQFLLFCHISSHQHDGMEA	a1	/
P20-39	ATRRYYLGAVELSWDYMQSD	A1	/
P178-197	LSHVDLVKDLNSGLIGALLV	A1	(13)
P1940-1959	INGYIMDTLPGLVMAQDQRI	A3	/
P2084-2103	KEPFSWIKVDLLAPMIIHGI	C1	(65)
P2161-2180	PPIIARYIRLHPTHYSIRST	C1/C2	(70)
P2226-2245	KARLHLQGRSNAWRPQVNNP	C2	(13, 61–65)
P2328-2347	HPQSWVHQIALRMEVLGCEA	C2	/
P1715-1734	RHYFIAAVERLWDYGMSSSP	A3	/
P1986-2005	KKEEYKMALYNLYPGVFETV	A3	/
P2068-2087	KLARLHYSGSINAWSTKEPF	C1	/
P2114-2133	SLYISQFIIMYSLDGKKWQT	C1	(66–69)
P2264-2283	TQGVKSLLTSMYVKEFLISS	C2	/
P2320-2339	LLTRYLRIHPQSWVHQIALR	C2	(13, 61, 64, 70)

In this study, overall, 29 healthy donors were tested *in vitro* for rFVIII T cell reactivity, and all of them gave rise to a specific T cell response. It remains to be unraveled which are the mechanisms of tolerance that counteract this self-reactivity under physiological conditions. The balance between self-reactivity and tolerogenic counterpart, which might be lacking during inhibitor development in HA patients, may also be the explanation for why certain individuals do develop inhibitors and others do not. In the previous study, FVIII-specific T cells originated equivalently from the naïve and memory repertoire, suggesting that part of the population has converted into memory phenotype, upon FVIII recognition in circulation, but did not expand (19). The detection of a specific FVIII regulatory T cell population has never been reported yet, but it has been suggested on healthy donors (71), and an enhanced FVIII T cell response was shown when the CD25+ fraction was depleted from total PBMCs (72). The isolation of naive T regulatory cells specific for FVIII could be further evidence of an active FVIII thymic selection as well as an explanation of the tolerogenic counterpart that seems to downregulate these auto-reactive T cell populations under homeostatic conditions.

Our work contributes to paving the way toward new and more conscious strategies of tolerance induction—for instance, immunodominant epitopes could be used as valid targets for FVIII CAR-Treg therapies, peptide-based tolerizing vaccines, or further deimmunization of rFVIII protein as previously attempted (64). Moreover, understanding the immune mechanism behind FVIII immunogenicity could help improve our understanding of the central tolerance mechanism to self.

## Data availability statement

All data are accessible on the NCBI GEO database, accession number: GSE253521 (https://www.ncbi.nlm.nih.gov/geo/query/acc.cgi?acc=GSE253521).

## Ethics statement

The studies involving humans were approved by Etablissement Français du Sang, Rungis, France. The studies were conducted in accordance with the local legislation and institutional requirements. The participants provided their written informed consent to participate in this study.

## Author contributions

VP: Conceptualization, Data curation, Formal analysis, Investigation, Methodology, Project administration, Software, Visualization, Writing – original draft, Writing – review & editing. GL: Data curation, Investigation, Methodology, Writing – review & editing. RG: Data curation, Formal analysis, Investigation, Software, Visualization, Writing – review & editing. EC: Investigation, Writing – review & editing. BM: Conceptualization, Funding acquisition, Methodology, Resources, Supervision, Validation, Writing – original draft, Writing – review & editing.
