# Supplementary material for: The self-reactive FVIII T cell repertoire in healthy individuals relies on a short set of epitopes and public clonotypes
Source: Front Immunol. 2024 Mar 6;15:1345195. doi: 10.3389/fimmu.2024.1345195 (PMC10951066; doi:10.3389/fimmu.2024.1345195)
Supplement: Supplementary file 1 [file Table_1.docx]

**Supplementary table ST1: HLA-DR restricted-epitopes retrieved from in silico prediction**

We performed *in silico* prediction analyses on the whole sequence of full-length FVIII, to determine a group of 9-mer cores with the highest binding score affinity for restricted HLA-DR alleles, selected as representative of the most expressed in the European and North American population. A public algorithm NetMHCpan 4.0 was used as a prediction tool to determine 63 20-mer length FVIII peptides, for their ability to bind promiscuously, with a percentile score below 10%, at least four HLA class II molecules in our panel. Therefore obtaining a library of peptides that span the total protein sequence, counts for more than 50% of its aa coverage. HLA-DR alleles panel used on NetMHCIIpan 4.0: HLA-DRB1*01:01, HLA-DRB1*03:01, HLA-DRB1*04:01, HLA-DRB1*04:05, HLA-DRB1*07:01, HLA-DRB1*08:02, HLA-DRB1*09:01, HLA-DRB1*11:01, HLA-DRB1*12:01, HLA-DRB1*13:01, HLA-DRB1*15:01, HLA-DRB3*01:01, HLA-DRB3*02:02, HLA-DRB4*01:01, HLA-DRB5*01:01. Details of the prediction are presented in table S6 (ST6)

| **Peptide pool** | **Residue numbers** | **Domain** | **Peptide sequence** |
| --- | --- | --- | --- |
| 1 | P11-30 | A1 | LCLLRFCFSATRRYYLGAVE |
|  | P20-39 | A1 | ATRRYYLGAVELSWDYMQSD |
|  | P75-94 | A1 | DHLFNIAKPRPPWMGLLGPT |
|  | P100-119 | A1 | YDTVVITLKNMASHPVSLHA |
|  | P178-197 | A1 | LSHVDLVKDLNSGLIGALLV |
|  | P285-304 | A1 | VHSIFLEGHTFLVRNHRQAS |
|  | P292-311 | A1 | GHTFLVRNHRQASLEISPIT |
|  | P307-326 | A1 | ISPITFLTAQTLLMDLGQFL |
|  | P322-341 | A1 | LGQFLLFCHISSHQHDGMEA |
| 2 | P383-402 | A2 | NSPSFIQIRSVAKKHPKTWV |
|  | P477-496 | A2 | GDTLLIIFKNQASRPYNIYP |
|  | P496-515 | A2 | PHGITDVRPLYSRRLPKGVK |
|  | P548-567 | A2 | LTRYYSSFVNMERDLASGLI |
|  | P582-601 | A2 | GNQIMSDKRNVILFSVFDEN |
|  | P601-620 | A2 | NRSWYLTENIQRFLPNPAGV |
|  | P607-626 | A2 | TENIQRFLPNPAGVQLEDPE |
|  | P629-648 | A2 | ASNIMHSINGYVFDSLQLSV |
|  | P652-671 | A2 | EVAYWYILSIGAQTDFLSVF |
| 3 | P668-687 | A2 | LSVFFSGYTFKHKMVYEDTL |
|  | P693-712 | A2 | SGETVFMSMENPGLWILGCH |
|  | P715-734 | a2 | DFRNRGMTALLKVSSCDKNT |
|  | P742-761 | B | YEDISAYLLSKNNAIEPRSF |
|  | P870-889 | B | PESGLQLRLNEKLGTTAATE |
|  | P890-909 | B | LKKLDFKVSSTSNNLISTIP |
|  | P1006-1025 | B | DNALFKVSISLLKTNKTSNN |
|  | P1011-1030 | B | KVSISLLKTNKTSNNSATNR |
|  | P1056-1075 | B | TEFKKVTPLIHDRMLMDKNA |
| 4 | P1065-1084 | B | IHDRMLMDKNATALRLNHMS |
|  | P1112-1131 | B | DMSFFKMLFLPESARWIQRT |
|  | P1157-1176 | B | VEGQNFLSEKNKVVVGKGEF |
|  | P1173-1192 | B | KGEFTKDVGLKEMVFPSSRN |
|  | P1191-1210 | B | RNLFLTNLDNLHENNTHNQE |
|  | P1239-1258 | B | TKNFMKNLFLLSTRQNVEGS |
|  | P1323-1342 | B | QNFVTQRSKRALKQFRLPLE |
|  | P1359-1378 | B | WSKNMKHLTPSTLTQIDYNE |
|  | P1407-1426 | B | LPIAKVSSFPSIRPIYLTRV |
| 5 | P1414-1433 | B | SFPSIRPIYLTRVLFQDNSS |
|  | P1423-1442 | B | LTRVLFQDNSSHLPAASYRK |
|  | P1484-1503 | B | SATNSVTYKKVENTVLPKPD |
|  | P1508-1527 | B | SGKVELLPKVHIYQKDLFPT |
|  | P1562-1581 | B | KVPFLRVATESSAKTPSKLL |
|  | P1715-1734 | A3 | RHYFIAAVERLWDYGMSSSP |
|  | P1724-1743 | A3 | RLWDYGMSSSPHVLRNRAQS |
|  | P1787-1806 | A3 | EDNIMVTFRNQASRPYSFYS |
|  | P1798-1817 | A3 | ASRPYSFYSSLISYEEDQRQ |
| 6 | P1931-1951 | A3 | FKENYRFHAINGYIMDTLPG |
|  | P1940-1959 | A3 | INGYIMDTLPGLVMAQDQRI |
|  | P1947-1966 | A3 | TLPGLVMAQDQRIRWYLLSM |
|  | P1956-1975 | A3 | DQRIRWYLLSMGSNENIHSI |
|  | P1986-2005 | A3 | KKEEYKMALYNLYPGVFETV |
|  | P2068-2087 | C1 | KLARLHYSGSINAWSTKEPF |
|  | P2084-2103 | C1 | KEPFSWIKVDLLAPMIIHGI |
|  | P2097-2116 | C1 | PMIIHGIKTQGARQKFSSLY |
|  | P2105-2124 | C1 | TQGARQKFSSLYISQFIIMY |
| 7 | P2114-2133 | C1 | SLYISQFIIMYSLDGKKWQT |
|  | P2161-2180 | C2 | PPIIARYIRLHPTHYSIRST |
|  | P2170-2189 | C2 | LHPTHYSIRSTLRMELMGCD |
|  | P2212-2231 | C2 | SSYFTNMFATWSPSKARLHL |
|  | P2226-2245 | C2 | KARLHLQGRSNAWRPQVNNP |
|  | P2264-2283 | C2 | TQGVKSLLTSMYVKEFLISS |
|  | P2276-2295 | C2 | VKEFLISSSQDGHQWTLFFQ |
|  | P2320-2339 | C2 | LLTRYLRIHPQSWVHQIALR |
|  | P2328-2347 | C2 | HPQSWVHQIALRMEVLGCEA |

**Supplementary table ST2. HLA haplotypes of all donors tested.**

| Exepriments | Donors | HLA-DR | | HLA-DP | |
| --- | --- | --- | --- | --- | --- |
| **FVIII-Ova** | 1179 | 14:54:01 | 15:01:01 | 02:01:02 | 03:01:01 |
|  | 1207 | 03:01:01 | 13:02:01 | 01:01:01 | 04:01:01 |
|  | 1195 | 03:01:01 | 11:01:01 | 02:01:02 | 14:01:01 |
|  | 1180 | 04:01:01 | 13:01:01 | 03:01:01 | 04:01:01 |
| **FVIII/18 epitopes** | 1327 | 11:01:01 | 15:01:01 | 02:01:02 | 04:01:01 |
|  | 1255 | 04:01:01 | 04:05:01 | 02:01:02 | 03:01:01 |
|  | 1238 | 03:01:01 | 15:01:01 | 01:01:01 | 02:01:02 |
|  | 1285 | 07:01:01 | 15:01:01 | 04:01:01 | 04:01:01 |
|  | 1245 | 07:01:01 | 10:01:01 | 01:01:01 | 04:01:01 |
| **epitope mapping** | 1217 | 01:01:01 | 07:01:01 | 02:01:02 | 04:01:01 |
|  | 1211 | 04:01:01 | 13:01:01 | 04:01:01 | 19:01:01 |
|  | 1218 | 01:01:01 | 01:01:01 | 02:01:02 | 04:01:01 |
|  | 1219 | 09:01:01 | 13:01:01 | 13:01:01 | 17:01:01 |
|  | 1212 | 04:01:01 | 10:01:01 | 02:01:02 | 04:01:01 |
|  | 1214 | 13:01:01 | 13:03:01 | 02:01:02 | 04:01:01 |
|  | 1257 | 16:01:01 | 07:01:01 | 02:01:02 | 04:01:01 |
|  | 1258 | 14:01:02 | 07:01:01 | 04:01:01 | 17:01:01 |
|  | 1223 | 13:01:01 | 13:02:01 | 02:01:02 | 05:02:01 |
|  | 1226 | 08:02:01 | 01:02:01 | 02:01:02 | 02:01:01 |
|  | 1244 | 06:02:01 | 11:01:01 | 03:01:01 | 04:01:01 |
|  | 1245 | 16:01:01 | 07:01:01 | 01:01:01 | 04:01:01 |
|  | 1224 | 04:01:01 | 15:01:01 | 04:01:01 | 04:01:01 |
|  | 1238 | 07:01:01 | 03:01:01 | 01:01:01 | 02:01:01 |
|  | 1260 | 07:02:01 | 01:01:01 | 04:01:01 | 04:02:01 |
|  | 1105 | 01:01:01 | 03:01:01 | 04:01:01 | 04:01:01 |
| **anti-HLA** | 1292 | 11:01:01 | 13:02:01 | 02:01:02 | 04:01:01 |
|  | 1289 | 08:02:01 | 11:03:01 | 03:01:01 | 04:01:01 |
|  | 1314 | 04:05:01 | 13:02:01 | 02:01:02 | 03:01:01 |
|  | 1313 | 01:02:01 | 03:01 | 01:01:01 | 04:02:01 |

**Supplementary Table ST3.**

**18 immunodominant T cell epitopes in healthy individuals.** Summary of data obtained for the FVIII-peptides identified as immunodominant T cell epitopes through a large-scale epitope mapping. The T cell responses to all 63 peptides tested were initially ranked based on donor coverage, which is defined as the number of donors generating a T cell response to each peptide. The top 10 peptides achieved 100% donor coverage, eliciting a response in all 16 tested donors. The response was further ranked based on the total number of positive T cell lines detected for each peptide across all donors (response intensity). To quantify the cumulative response intensity, we calculated the percentage of the total number of FVIII T cell lines detected, namely 872 FVIII-specific T cell lines. Using this dual ranking approach, we identified 18 FVIII peptides responsible for 100% of donors coverage and approximately 50% of the total response to rFL-FVIII.

| **Position** | **Sequence** | **FVIII Domain** | **responding donors** | **Donor coverage**  **( %)** | **cumulativedonor coverage**  **(%)** | **nb positive T cell lines** | **response intensity**  **( %)** | **Cumulative response intensity**  **(%)** |
| --- | --- | --- | --- | --- | --- | --- | --- | --- |
| P285-304 | **VHSIFLEGHTFLVRNHRQAS** | A1 | 12 | 75 | 75 | 44 | 5,05 | 5,05 |
| P292-311 | **GHTFLVRNHRQASLEISPIT** | A1 | 12 | 75 | 81 | 26 | 2,98 | 8,03 |
| P75-94 | **DHLFNIAKPRPPWMGLLGPT** | A1 | 11 | 69 | 81 | 38 | 4,36 | 12,39 |
| P100-119 | **YDTVVITLKNMASHPVSLHA** | A1 | 11 | 69 | 81 | 44 | 5,05 | 17,43 |
| P322-341 | **LGQFLLFCHISSHQHDGMEA** | a1 | 10 | 62 | 81 | 27 | 3,10 | 20,53 |
| P20-39 | **ATRRYYLGAVELSWDYMQSD** | A1 | 10 | 62 | 81 | 43 | 4,93 | 25,46 |
| P178-197 | **LSHVDLVKDLNSGLIGALLV** | A1 | 9 | 56 | 81 | 19 | 2,18 | 27,64 |
| P1940-1959 | **INGYIMDTLPGLVMAQDQRI** | A3 | 9 | 56 | 94 | 14 | 1,61 | 29,24 |
| P2084-2103 | **KEPFSWIKVDLLAPMIIHGI** | C1 | 9 | 56 | 94 | 20 | 2,29 | 31,54 |
| P2161-2180 | **PPIIARYIRLHPTHYSIRST** | C1/C2 | 9 | 56 | 100 | 21 | 2,41 | 33,94 |
| P2226-2245 | **KARLHLQGRSNAWRPQVNNP** | C2 | 9 | 56 | 100 | 12 | 1,38 | 35,32 |
| P2328-2347 | **HPQSWVHQIALRMEVLGCEA** | C2 | 9 | 56 | 100 | 23 | 2,64 | 37,96 |
| P1715-1734 | **RHYFIAAVERLWDYGMSSSP** | A3 | 8 | 50 | 100 | 16 | 1,83 | 39,79 |
| P1986-2005 | **KKEEYKMALYNLYPGVFETV** | A3 | 8 | 50 | 100 | 18 | 2,06 | 41,86 |
| P2068-2087 | **KLARLHYSGSINAWSTKEPF** | C1 | 8 | 50 | 100 | 20 | 2,29 | 44,15 |
| P2114-2133 | **SLYISQFIIMYSLDGKKWQT** | C1 | 8 | 50 | 100 | 10 | 1,15 | 45,30 |
| P2264-2283 | **TQGVKSLLTSMYVKEFLISS** | C2 | 8 | 50 | 100 | 12 | 1,72 | 47,02 |
| P2320-2339 | **LLTRYLRIHPQSWVHQIALR** | C2 | 8 | 50 | 100 | 10 | 1,15 | 48,17 |

**Supplementary table ST4.**

**HLA restriction and FVIII specificity.**

Four healthy donors were used as the source of CD4 T cells, which were amplified *in vitro* with the pool of 18 FVIII peptides selected for study. In an initial IFN-γ ELISPOT assay, we screened each of the 18 peptides individually to identify any positive T cell responses to both the full-length FVIII protein (protein specificity) as well as the specific peptide inducing the response. In a second IFN-γ ELISPOT assay, we tested the same T cell line against the respective positive peptide alone and with the anti-HLA-DP (B7/21), anti-HLA-DQ (SPVL3), or anti-HLA-DR (L243) as four independent conditions. Restriction was confirmed when inhibition was higher than 50% of the ELISPOT count compared with the peptide only. Data were reported as the percentage of HLA-, –DQ and -DP restricted T cell lines specific for each peptide.

| **Peptide** | **Responding donors**  **(%)** | **intensity (%)** | **HLA restriction (%)** | | | **Protein specificty** |
| --- | --- | --- | --- | --- | --- | --- |
|  |  |  | **DR** | **DP** | **DQ** |  |
| **P20-39** | 50 | 4,6 | 63 | 37 | - | **+** |
| **P75-94** | 25 | 1,1 | - | 100 | - | **+** |
| **P2328-2347** | 25 | 1,1 | 100 | - | - | **+** |
| **P1986-2005** | 25 | 1,1 | 100 | - | - | **+** |
| **P322-341** | 50 | 4,6 | 100 | - | - | **+** |
| **P2084-2103** | 100 | 19,5 | 53 | 47 | - | **+** |
| **P285-304** | 50 | 3,4 | 100 | - | - | **+** |
| **P1940-1959** | 25 | 1,1 | 100 | - | - | - |
| **P2226-2245** | 50 | 12,6 | 77 | 23 | - | **+** |
| **P100-119** | 50 | 8 | 100 | - | - | **+** |
| **P178-197** | 75 | 4,6 | 50 | 50 | - | **+** |
| **P292-311** | - | - | - | - | - | - |
| **P1715-1734** | 75 | 18,4 | 100 | - | - | **+** |
| **P2068-2087** | 25 | 1,1 | 100 | - | - | - |
| **P2114-2133** | 50 | 3,4 | 66 | 33 | - | **+** |
| **P2161-2180** | 75 | 9,2 | 81 | 19 | - | **+** |
| **P2264-2283** | - | - | - | - | - | - |
| **P2320-2339** | 25 | 2,3 | 75 | 25 | - | **+** |

**Supplementary table ST5. HLA class II binding assay**

Quantitative peptide-MHCII competition binding assays were carried out to determine binding avidities of the 18 20-mer FVIII peptides to 6 HLA-DRB1. Data are presented as ratio between reference peptide IC50/FVIII-peptide IC50 tested. Briefly, non-biotinylated FVIII peptides have been diluted at a concentration of 20 mM in DMSO. 10-µl aliquots of serially diluted (90.000nM – 0.9nM in n-Octyl b-D-Glucopyranoside, 2.5 mg/ml in 1x phosphate buffer, pH 5.9) of non-biotinylated FVIII peptides, were added to duplicate wells of 96-well polypropylene plates. Serially diluted non-FVIII reference peptides known to bind to the specific HLA-DRB1 were added to separate wells as positive controls. Next, the biotinylated peptide at a concentration of 2mM was use to prepare 1000-fold pre-dilution to reach a final concentration of 15nM. The HLA-DRB1 proteins were diluted to 30 nM in n-Octyl β-D-Glucopyranoside, 2.5 mg/ml in 1x phosphate buffer, pH 5.9. The anti-HLA-DR antibody L243 (5 μg/mL in 12.5 mM borate buffer, pH 8.2) were added to 96-well ELISA plates, and the plates were incubated at 2-8°C overnight and blocked with 1xPBS containing 5% FCS and 0.1% NAN3. SA-HRP (diluted 5000x in 1xPBS 0.1% Tween20) was added to each well. The plates were covered and incubated at room temperature for 60 min at 2-8°C and washed. TMB (100 μl/well) was then added, and the plates were incubated at room temperature for 15-30 min. ELISA stopping solution (0.5M H2SO4) was added and then read on a FLUOstar OPTIMA fluorometer. Sigmoidal binding curves were simulated and IC50 values calculated for the FVIII peptides, based on their competition with the reference peptides for each HLA-DRB1. Data were reported as the ratio between the values of the tested peptide and of the reference peptide. Ratios below 10 correspond to good biding affinity.

|  | Ratio IC50 | | | | | |
| --- | --- | --- | --- | --- | --- | --- |
| **FVIII peptide** | **DRB1*0101** | **DRB1*0301** | **DRB1*0401** | **DRB1*0701** | **DRB1*1101** | **DRB1*1501** |
| **P20-39** | NA | 850 | 1.7 | 0.5 | 0.8 | NA |
| **P75-94** | 747 | 40 | 27 | 1 | 1.8 | 0.0 |
| **P2328-2347** | 1.6 | 0.0 | 1240 | 1 | 2 | NA |
| **P1986-2005** | 182 | NA | 622.5 | 0.2 | NA | 0.4 |
| **P322-341** | NA | NA | NA | 0.6 | 0.05 | 0.05 |
| **P2084-2103** | NA | 6.6 | 1.9 | NA | NA | NA |
| **P285-304** | 163 | 58 | 57.6 | 2 | 0.0 | 11 |
| **P1940-1959** | 6 | NA | 0.1 | 1.1 | 0.5 | NA |
| **P2226-2245** | 0.2 | 33 | 3 | 0.05 | 1.9 | 0.6 |
| **P100-119** | 226 | 30 | 17 | 3 | 2 | 0.5 |
| **P178-197** | 1.7 | 0.05 | 0.05 | 0.4 | 0.1 | 95 |
| **P292-311** | 34 | 72 | 22.5 | 1 | 0.05 | NA |
| **P1715-1734** | 20.4 | NA | 138 | 1 | NA | 77 |
| **P2068-2087** | 70.5 | NA | 0.2 | 0.35 | 4.6 | 20 |
| **P2114-2133** | 0.2 | 30 | 0.6 | 0.1 | 0.4 | 4 |
| **P2161-2180** | 1 | 21 | 5.6 | 0.05 | 0.4 | 0.05 |
| **P2264-2283** | 6242 | 17 | 5.5 | 0.05 | 0.05 | 480 |
| **P2320-2339** | 1 | 114 | 76 | 0.05 | 1 | 0.0 |
| **Reference peptide:** | **PKYVKKQNTLKLAT** | **SFLLTDLINRRTPRVDGQ** | **PKYVKKQNTLKLAT** | **PVDLHAFLSQAVFS** | **PKYVKKQNTLKLAT** | **ENPVVHFFKNIVTPR** |

Table ST6: percentile scores of the 18 immunoprevalent T cell epitopes

*in silico* prediction analyses were carried out using NetMHCpan 4.0 by submiiting the whole sequence of full-length FVIII to the algorithm. The data presented in this supplementary detailed the HLA binding scores (percentiles) of the 9-mer cores comprised in the of the selected 18 peptides.
